# Application of Artificial Intelligence to Assess the Risks of Simultaneous Operations for Patients with Concomitant Atherosclerotic Damage of Coronary and Carotid Arteries

**DOI:** 10.17691/stm2022.14.1.06

**Published:** 2022-01-28

**Authors:** L.N. Ivanov, V.G. Petrenko, N.I. Grishina, А.S. Mukhin

**Affiliations:** Professor, Department of Hospital Surgery named after B.A. Korolyov; Privolzhsky Research Medical University, 10/1 Minin and Pozharsky Square, Nizhny Novgorod, 603005, Russia; Cardiovascular Surgeon; Specialized Cardiosurgical Clinical Hospital named after Academician B.A. Korolev, 209 Vaneeva St., Nizhny Novgorod, 603136, Russia; Cardiovascular Surgeon; Specialized Cardiosurgical Clinical Hospital named after Academician B.A. Korolev, 209 Vaneeva St., Nizhny Novgorod, 603136, Russia; Clinical Resident; Privolzhsky Research Medical University, 10/1 Minin and Pozharsky Square, Nizhny Novgorod, 603005, Russia;; Professor, Head of the Department of Hospital Surgery named after B.A. Korolyov; Privolzhsky Research Medical University, 10/1 Minin and Pozharsky Square, Nizhny Novgorod, 603005, Russia;

**Keywords:** multifocal atherosclerosis, artificial intelligence, internal carotid artery, coronary arteries

## Abstract

**The aim of the study** is to assess the possibility of using artificial intelligence to determine the most significant predictors of the operative correction outcomes for patients with damaged coronary and carotid arteries.

**Materials and Methods:**

The retrospective study of the simultaneous (or single-stage) surgical intervention results has been carried out in patients with combined atherosclerotic damage of the coronary bed and cerebral arteries (n=42), which was severe and extensive. The parameters which may be predictors of the cardiovascular risk were analyzed using the TADA program. Ten models were built for program learning. The model with 92% predictive accuracy appeared to be the most successful.

**Results:**

Simultaneous correction resulted in the absence of 30-day coronary complications in all patients. With respect to the cerebral vascular territory, acute ischemic stroke developed in 2 patients. The lethality rate was 2.4%, the fatal outcome was caused by postoperative gastrointestinal bleeding.

The TADA program model considered the following parameters to be the most significant predictors: internal carotid artery cross-clamping time in minutes (51.24%); damage to the left coronary artery stem (30.42%); diastolic AP (18.28%). If cross-clamping of the internal carotid artery lasts for less than 18 min, complications are not likely to occur, while they are practically inevitable if the time exceeds 46 min. The probability of complications grows nonlinearly with the increase of the extent of the left coronary artery stem injury. A high diastolic AP never virtually coincides with the presence of complications, nor does the low one. The highest probability of complications is at the values from 70 to 80 mm Hg.

In patients with a triple vessel injury of the coronary arteries, a representative picture of a nonsignificant feature is observed.

**Conclusion:**

Application of artificial intelligence for determining risk predictors for patients with concurrent atherosclerotic damage of the coronary and carotid arteries is an effective method for prognosticating the risks of simultaneous interventions.

## Introduction

In cardiovascular surgery, the most frequent event in patients is multifocal atherosclerosis associated with formation of dangerous hemodynamically significant damages in the coronary and carotid arteries [1]. The concomitant presence of atherosclerotic injury in more than two vascular areas is connected with the risk of myocardial infarction (MI), acute cerebrovascular accident (ACVA), and fatal outcome which creates difficulties in choosing the optimal management strategy. In order to minimize the risk of developing neurological and coronary complications, simultaneous or staged operations are performed, the tactics of which are based on the myocardial and brain functional reserves [2]. Staged treatment of patients with multiple concomitant pathologies has some drawbacks: two operative interventions and anesthetic supports, long hospital stay, and a high risk of complications in the intact area at the first stage of surgical treatment [3–5]. The advantage of the simultaneous operations is one anesthetic support and shorter time of the postoperative rehabilitation period [6]; however, there remains the risk connected with a large hemodynamic load and duration of perfusion provision [7, 8]. In simultaneous operations, blood flow in the coronary and carotid arteries may be restored in early terms excluding the development of complications which could occur in staged operations in the intact territory [9]. Nevertheless, the assessment of simultaneous revascularization safety requires further investigation. Implementation of mathematic methods of artificial intelligence, creation of a prognostic model based on the patient risk predictor analysis opens additional possibilities for choosing optimal strategies of managing patients with concomitant injury of coronary and carotid arteries and personified simultaneous revascularization.

**The aim of the study** is to assess the possibility of using artificial intelligence to determine the most significant predictors of the operative correction outcomes for patients with damaged coronary and carotid arteries.

## Materials and Methods

The results of treatment of 178 patients with concurrent ischemic heart (IHD) and brain diseases have been analyzed retrospectively. Treatment was conducted at the Specialized Cardiosurgical Clinical Hospital named after Academician B.A. Korolev (Nizhny Novgorod, Russia) in the period from 2011 to 2019. 42 patients underwent a single-stage reconstruction of the coronary and carotid arteries. Coronary and carotid artery damages in patients with simultaneous carotid endarterectomy (CE) and aortocoronary bypass (ACB) were, as a rule, severe and extensive. 136 patients with concomitant coronary and carotid artery injury were performed open staged operations and endovascular interventions as one of the revascularization stages. These patients were excluded from the study since pure comparison of the two strategies is not always possible.

The study complied with the Declaration of Helsinki (2013) and was approved by the Ethics Committee of Privolzhsky Research Medical University (Nizhny Novgorod, Russia). Written informed consent was obtained from each patient.

The clinical characteristic of the tested patients is presented in Table 1. Men composed the majority of them. The body mass index was slightly increased.

**Table 1:** Clinical characteristics of patients with coronary and carotid artery injury (n=42)

Description of patients	Value	95% CI
Age (years), М±σ	63±7	61–65
Sex, n (%):		
male	37 (88)	
female	5 (12)	
Body mass index >25, М±σ	26.7±3.6	25.6–27.8
NYHA, n (%):		
II	8 (19.0)	
III	31 (73.8)	
IV	3 (7.1)	
Prior myocardial infarction, n (%)	25 (59.5)	—
Unstable angina, n (%)	2 (4.8)	—
Left ventricular ejection fraction, <60% (%), М±σ	56.9±9.1	54.2–59.7
End-diastolic volume (ml), М±σ	103.3±32.2	93.5–113.1
End-systolic volume (ml), М±σ	45.8±24.1	38.5–53.1
Degree of chronic cerebrovascular insufficiency, n (%):		
grade 1	32 (76.2)	
grade 2	2 (4.8)	
grade 3	3 (7.1)	
grade 4	5 (11.9)	
Type 2 diabetes mellitus, n (%)	11 (26.2)	—
Chronic renal failure, n (%)	3 (7.1)	—

Coronary insufficiency implied the distribution of patients according to angina functional classification (FC) and the number of the damaged coronary arteries. More than half of the patients had MI in the past history. The degree of chronic cerebrovascular insufficiency was defined according to A.V. Pokrovsky classification (1979): asymptomatic course prevailed in the overwhelming majority.

The risk of cardiosurgical interventions was assessed using the EuroSCORE classification based on the severity of the comorbid condition: a low risk (score 0–2) was observed in 17 patients (95% CI: 1.81–2.38); moderate risk (score 3–5) in 9 patients (95% CI: 3.79–4.71); high risk (score 6–45) in 16 (95% CI: 5.39–9.78). An average value of the EuroSCORE coefficient was 4.45%.

The degree of carotid artery damage was assessed by the data of duplex scanning with color mapping. Atherosclerotic plaques of carotid localization were visualized according to C.M. Steffen and G. Geroulakos classification (1993): type I was observed in 2 (4.8%) patients; type II in 8 (19.0%); type III in 13 (30.9%); type IV in 11 (26.2%); type V in 8 (19.0%) patients. Atherosclerotic injury of the third area, i.e. the lower limb arteries, was found in 3 (7.1%) patients, infrarenal abdominal aortic aneurysm — in 1 (2.4%) patient. Arterial hypertension (AH) was noted in the majority of patients (57.1%). Isolated systolic AH was in 10 (24.0%) patients, while the rest suffered from a variable course of AH (Table 2).

**Table 2:** AP variability in patients with coronary and carotid artery injury (n=24)

Arterial hypertension	Systolic AP, 95% CI	Diastolic AP, 95% CI
Stage 1 (n=7)	138.44–152.99	90.22–93.50
Stage 2 (n=5)	164.89–187.10	99.38–106.62
Stage 3 (n=2)	141.47–268.53	51.46–178.53
Isolated systolic (n=10)	143.05–158.55	75.26–85.34

Simultaneous operations were performed on the patients with a reduced reserve of the coronary and cerebral circulation. The main indications to simultaneous operations were as follows: injury of the left coronary artery stem, triple vessel injury of the coronary bed, angina pectoris III–IV FC, unstable angina, a high-risk coefficient by the EuroSCORE scale, hemodynamically significant stenosis of the carotid artery, occlusions of the contralateral internal carotid artery (ICA), and bilateral carotid artery damage.

In case of the single-stage operations, the ICA was reconstructed first, and then revascularization of the coronary territory was performed using normothermic artificial blood circulation (AC) and crystalloid pharmaco-cold cardioplegia (Custodiol and Consol solutions).

Types of the single-stage operations performed are listed below:

eversion coronary endarterectomy — 12 (28.6%) patients;

classic coronary endarterectomy — 22 (52.4%) patients;

aorto-bicarotid bypass — 3 (7.1%) patients;

coronary endarterectomy with ICA plasty — 4 (9.5%) patients;

ICA redressment and resection — 1 (2.4%) patient; right ICA replacement — 2 (4.8%) patients;

АCB — 42 (100%) patients.

The main indices of the intraoperative period (М±σ): aorta cross-clamping — 68.2±53.5 min;

ICA cross-clamping — 34.4±10.5 min;

perfusion — 87.1±29.8 min;

perfusion volume — 1142.9±259.6 min.

The tested parameters were loaded into the TADA program. Ten models were built for learning. The accuracy was assessed for each model. The model with 92% predictive accuracy appeared to be the most successful. Let us consider it in more detail. All data in this model were divided into three parts: 40%; 30%; 30%. The first subset was called “training”, the second — “validation”, the third — “test”. By means of the training sample, the program selected 10,000 formulas which had the potential to predict well the target variable on the basis of the non-target ones. The second subset allowed the selected formulas to compete with each other and to choose one the most successful. The third subset used this formula to predict the diagnosis and generate indices of accuracy.

The quality of prediction was estimated by the following characteristics: predictive accuracy, Matthews correlation coefficient (MCC); area under the curve (AUC); true positive rate (TPR): the proportion of the accurately identified positive outcomes to the total number of positive outcomes; true negative rate (TNR): the proportion of the accurately assessed negative outcomes to the total number of negative outcomes; positive predictive value (PPV): the proportion of the accurately assessed positive outcomes to the total objects which were marked by the model as positive.

Thus, a harmonic mean for the two values, precision (PPV) and completeness (recall), is calculated by the formula: where *tp is* the number of true positives; *fp* is the number of false positives; *fn* is the number of false negatives.


$$F1=\frac{\text{2}}{{\text{recall}}^{-1}{\text{ +precision}}^{-1}}=2\frac{\text{precision recall}}{\text{precision +recall}}=\frac{tp}{tp\text{+}\frac{1}{2}{\left(fp+fn\right)}^{\text{,}}}$$

where *tp* is the number of true positives; *fp* is the number of false positives; *fn* is the number of false negatives.

**Statistical data processing** was conducted concurrently with the data analysis in the TADA program using Microsoft Excel 2019 and Statistica 10.0 software. Distribution normality was checked by means of Kolmogorov–Smirnov test. Arithmetic mean (M) and standard deviation (σ) were calculated for the data following the law of normal distribution. The results of all tests were considered statistically significant at the value below critical (р≤0.05). The influence of the risk predictors was studied by their dependence on the postoperative complications; the Pearson correlation coefficient was calculated for the comparison.

## Results

Simultaneous correction resulted in the absence of 30-day coronary complications in all patients (n=42). On the part of the cerebrovascular area, 2 (4.8%) patients developed ACVA on the type of ischemic stroke: in one — in the contralateral area; in the second, thrombosis of ICA on the ipsilateral side was formed and the patient underwent thrombendarterectomy with the regress of the neurological symptoms. Lethal outcome was in 1 (2.4%) patient due to postoperative gastrointestinal bleeding. There were also nonspecific complications associated with their individual concurrent pathologies, specific character of the anesthesiological support, development of the reperfusion damage, electrolytic and metabolic disorders, usage of the tonic support, hemodynamic instability due to MI: exudative pleurisy in 2 (4.8%) patients, arrhythmias (paroxysmal supraventricular tachycardia, atrial fibrillation, in particular) in 4 (9.5%), pneumonia in 1 (2.4%).

**Figure 1:**
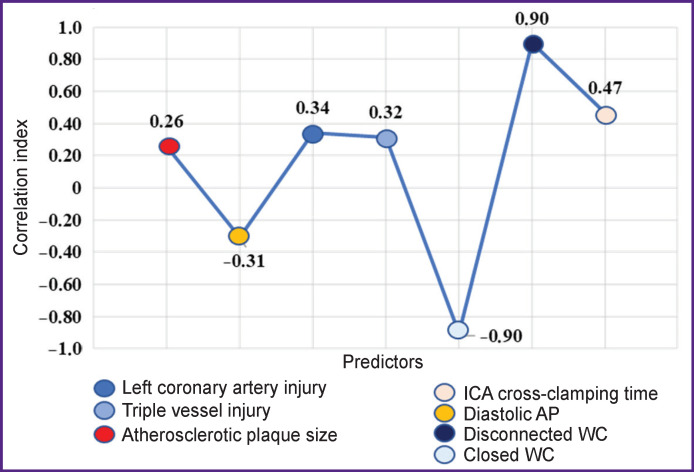
Risk predictors for postoperative complications

The data of the correlational study (Figure 1) demonstrate the significance of risk predictors in the development of cardiovascular complications. ICA cross-clamping has moderate positive correlation. Damage of the left coronary artery, triple vessel injury, and extension of the ICA atherosclerotic plaque showed weak positive correlation. The risk of postoperative complications increases with the reduction of the systolic AP: weak negative correlation.

The most significant risk predictors having a very high correlation are specific features of the Willis’ circle (WC). Disconnected WC increases the number of postoperative complications — positive relation, while closed WC, on the contrary, has a tendency to the decrease of the postoperative complication risk — negative relation.

**Figure 2:**
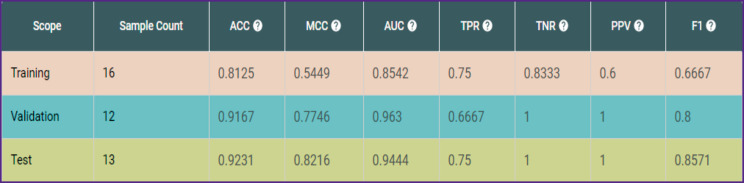
Parametric values for three subsets (data for 41 patients) Here: ACC — prediction accuracy; MCC — Matthews correlation coefficient; AUC — area under the curve; TPR — true positive rate (the proportion of the accurately identified positive outcomes to the total number of positive outcomes); TNR — true negative rate (the proportion of the accurately assessed negative outcomes to the total number of negative outcomes); PPV — positive predictive value (the proportion of the accurately assessed positive outcomes to the total objects which were marked by the model as positive); F1 — probability

The characteristics values in the three subsets calculated on the basis of the data from 41 patients were good, expected accuracy — 92%. MCC and AUC are rather high on the training subset, TPR (recall) is 0.75; PPV (precision) is 1 (Figure 2). This may be interpreted in the following way: the model does not actually make mistakes of the first kind, i.e. it does not raise the false alarm (if the model predicted complications with 100% probability, that’s actually how it is). On the other hand, there is a small probability that the model could miss a complicated case and predict a favorable outcome, but in reality, complications will occur anyway.

On the test and validation subsets, the model made one mistake of the second kind in each: it predicted absence of the complication, although it occurred.

**Figure 3:**
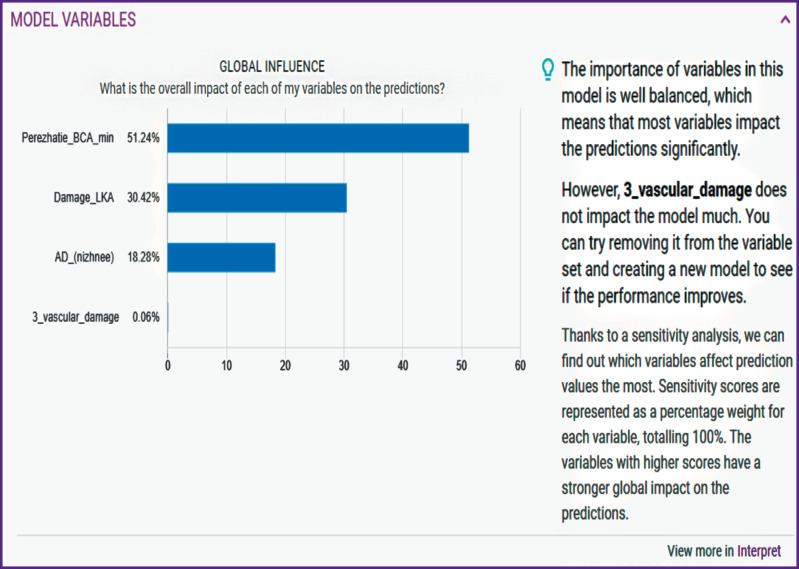
The most significant predictors of risk for development of postoperative complications

The greater part of the parameters was excluded as less significant at the first stage of data processing. The following predictors were considered by the model the most significant (in decreasing order): the time of ICA cross-clamping in minutes (51.24%); left coronary artery stem damage (30.42%); diastolic AP (18.28%) (Figure 3).

**Figure 4:**
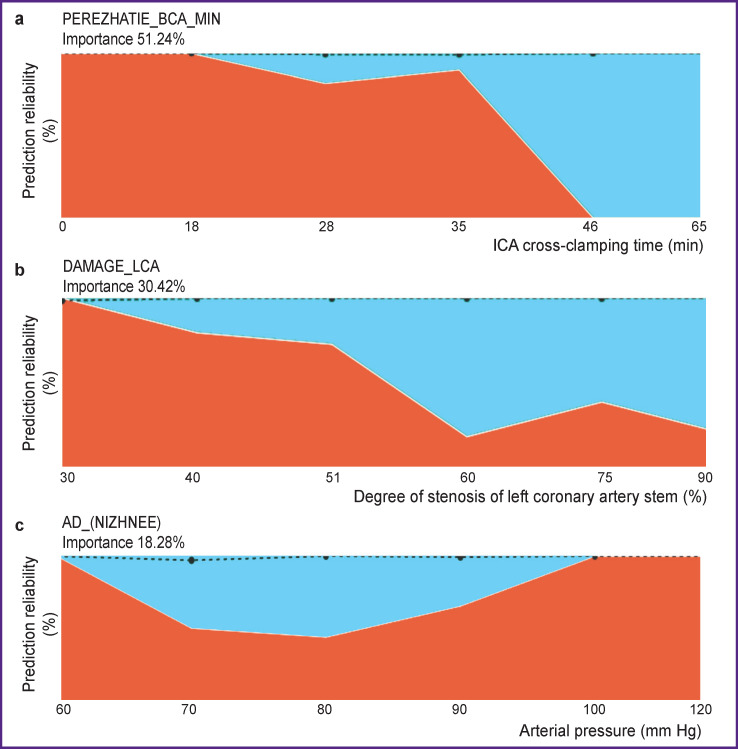
Degree of prediction reliability depending on: (а) time of internal carotid artery cross-clamping; (b) injury of left coronary artery stem; (c) arterial hypertension. Blue-colored area denotes complications, red is their absence

The degree of prediction reliability depending on the risk predictor value is shown in Figure 4. If the time of ICA cross-clamping is less than 18 min, there will be no complications, while complications are actually inevitable if the time exceeds 46 min (Figure 4 (а)).

The probability of complications increases nonlinearly with the increase of the ICA stem damage degree (Figure 4 (b)). Thus, according to our data, the probability of postoperative complications at 60% injury of the ICA stem is more than at 75% injury. Besides, if stenosis reaches 90%, the probability increases again as at 60%.

Neither high nor low diastolic AP coincides with the presence of complications. The greatest probability of complications is at the values from 70 to 80 mm Hg (Figure 4 (c)).

In the group of patients, who developed complications in the postoperative period, the diastolic AP was within 80–85 mm Hg. It is likely to be associated with the presence of isolated AH with unilateral systolic AP in this group of patients.

**Figure 5:**
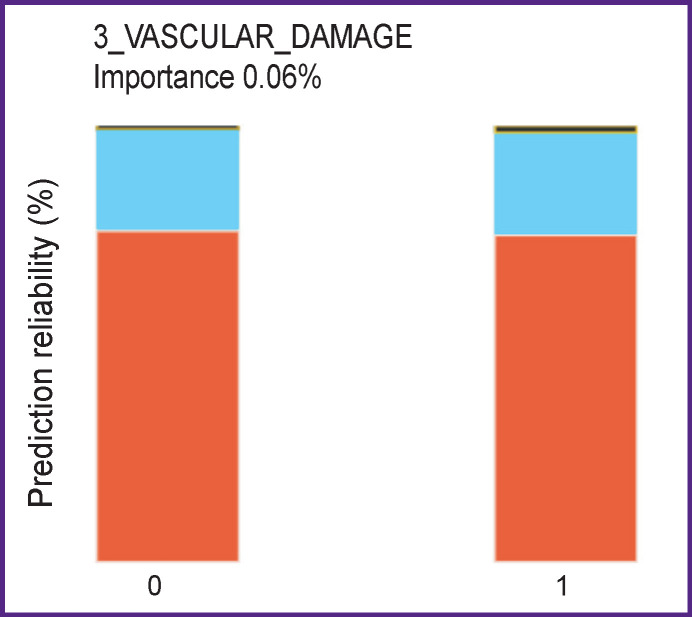
Triple vessel injury of coronary arteries is an insignificant feature depending on absence (*0*) or presence (*1*) of this parameter Blue-colored area denotes complications, red is their absence

In patients with a triple vessel coronary artery injury, a representative picture of insignificant feature was observed (Figure 5). Irrespective of the presence or absence of this parameter, it is impossible to predict complications, as the situations are essentially identical in both cases.

## Discussion

In our investigation, patients possessed a number of clinical and anatomic features. A high frequency (56.10%) of bilateral and multiple injuries of the brachiocephalic arteries is characteristic in this category of patients. A more severe clinical-angiographic picture of the coronary bed damage has been noted compared to other IHD cases and a more frequent involvement of the multivessel injury of the coronary arteries and left coronary artery stem in the process. More severe clinical manifestations of IHD associated with unstable angina, low myocardial contractility also attract attention. At the same time, in case of substantial reserve reduction of the coronary circulation and blood flow in the brachiocephalic arteries, simultaneous surgical operation on the two areas is preferable although it is connected with a high risk of complications. Prediction of complication risks acquires greater importance for making clinical decisions for this cohort of patients. Partially, this problem is solved by implementation of risk scales: SCORE, TIMI, CRUSADE, GRACE, etc. may serve as an example of their usage in cardiology and cardiovascular surgery [10, 11].

Risk predictors associated with the development of postoperative complications estimated in the course of statistical data processing were in line with the results obtained after data processing by the artificial intelligence.

Meyer et al. [12] have demonstrated the possibility of using deep machine learning methods to predict serious complications during real-time intensive therapy after open-heart cardiosurgical intervention. Data analysis of 9269 patients has shown that a diagnostic and prognostic model based on the machine learning algorithm is capable of improving absolute complication prediction by 29% for bleeding, 24% for mortality and renal failure against the clinical reference tools.

Presence of ischemic stroke in the outcomes of the cardiosurgical interventions was supposed to be connected with a number of factors. Prolonged ICA cross-clamping increases the risk of postoperative complications due to the development of cerebral ischemia. Cannulation of the aterosclerotically altered ascending aorta may lead to cerebral vessel embolism. The duration of MI elevates the risk of a stroke in patients with a disconnected WC and a bilateral carotid artery injury due to the insufficient compensation of the collateral blood circulation in the period of artificial perfusion. A surgical correction of the carotid artery may be a confounding factor. In our study, 2 (4.8%) patients were diagnosed with contralateral ICA occlusion. Occlusion of the contralateral ICA is a well-established predictor of the 30-day risk for a stroke or lethal outcome in patients with previous coronary endarterectomy [13].

The development of nonspecific postoperative complications (cardiac rhythm disturbance, pneumonia, exudative pleurisy) is supposed to be associated with ischemic and reperfusion injury during MI. The data obtained by us agree with the results of previous investigations [14, 15].

The obtained data on the risk predictors may be useful for primary selection of patients for cardiosurgical operations. The application of artificial intelligence in the cardiovascular practice will substantially help physicians at the multi-disciplinary case conference to define the surgical treatment tactics. However, the role of natural intelligence should not be excluded in the assessment of the clinical and somatic status of the patient and in making a decision whether a single-stage operation is indicated in each individual case.

### Study limitations

The sample used is too small to determine the probability of some undesirable event to occur. Further accumulation of clinical material to ascertain the expedience of the prognostic model application is necessary.

## Conclusion

The time of internal carotid artery cross-clamping, injury of the left coronary artery stem, disconnected Willis’ circle appeared to be the most significant predictors of the risk for simultaneous operations on the coronary and carotid arteries.

Application of artificial intelligence to determine the risk predictors in patients with concomitant damage of the coronary and carotid arteries is an effective method of predicting the risk for simultaneous cardiosurgical interventions in patients with multifocal atherosclerosis.
